# DGV: Dengue Genographic Viewer

**DOI:** 10.3389/fmicb.2016.00875

**Published:** 2016-06-07

**Authors:** Akifumi Yamashita, Tetsuya Sakamoto, Tsuyoshi Sekizuka, Kengo Kato, Tomohiko Takasaki, Makoto Kuroda

**Affiliations:** ^1^Pathogen Genomics Center, National Institute of Infectious DiseasesTokyo, Japan; ^2^Remote Operations Services Department, CTC System Management CorporationTokyo, Japan; ^3^Department of Virology I, National Institute of Infectious DiseasesTokyo, Japan

**Keywords:** NGS, dengue virus, genotyping, genograph, web service

## Abstract

Dengue viruses (DENVs) and their vectors are widely distributed throughout the tropical and subtropical regions of the world. An autochthonous case of DENV was reported in Tokyo, Japan, in 2014, for the first time in 70 years. A comprehensive database of DENV sequences containing both serotype and genotype data and epidemiological data is crucial to trace DENV outbreak isolates and promptly respond to outbreaks. We constructed a DENV database containing the serotype, genotype, year and country/region of collection by collecting all publically available DENV sequence information from the National Center for Biotechnology Information (NCBI) and assigning genotype information. We also implemented the web service Dengue Genographic Viewer (DGV), which shows the geographical distribution of each DENV genotype in a user-specified time span. DGV also assigns the serotype and genotype to a user-specified sequence by performing a homology search against the curated DENV database, and shows its homologous sequences with the geographical position and year of collection. DGV also shows the distribution of DENV-infected entrants to Japan by plotting epidemiological data from the Infectious Agents Surveillance Report (IASR), Japan. This overview of the DENV genotype distribution may aid in planning for the control of DENV infections. DGV is freely available online at: (https://gph.niid.go.jp/geograph/dengue/content/genomemap).

## Introduction

Dengue viruses (DENVs) are members of the genus *Flavivirus* in the family *Flaviviridae* and consist of four serotypes (DENV-1 to -4) (Lanciotti et al., 1992; Kuhn et al., 2002). Each serotype can be divided into five to six genotypes. However, there is no standard genotyping classification [DENV-1, (Goncalvez et al., 2002); DENV-2, (Anez et al., 2011; Khan et al., 2013); DENV-3, (Lanciotti et al., 1994; Wittke et al., 2002; Klungthong et al., 2008); DENV-4, (Abubakar et al., 2002)]. DENV has a positive-sense, single-stranded RNA genome that is ~11 kb in length and encodes a capsid protein (C), premembrane protein (prM), and envelope glycoprotein (E) in addition to seven non-structural proteins (NSs, NS1, NS2A, NS2B, NS3, NS4A, NS4B, and NS5). DENV infection causes dengue illness, which may range from dengue fever (a mild illness) to dengue hemorrhagic fever and dengue shock syndrome (the severe forms of the illness) in addition to asymptomatic cases (Centers for Disease Control and Prevention; http://www.cdc.gov/dengue/clinicalLab/clinical.html). Infection results in lifetime immunity against the same serotype, but successive exposure to different DENVs increases the likelihood of contracting a severe form of dengue illness, such as dengue hemorrhagic fever or dengue shock syndrome (Chiappelli et al., 2014).

DENV and its vectors have become widely distributed throughout the tropical and subtropical regions of the world (Murray et al., 2013). An autochthonous case of DENV infection was reported in Tokyo, Japan, in 2014 for the first time in 70 years (Kutsuna et al., 2015). To ensure prompt action in response to a DENV outbreak, a comprehensive DENV database based on the genotypes would be essential for tracing the outbreak source. To date, only two DENV databases provide genotype information. The web service ViPR^1^ (Pickett et al., 2012) supports genetic analysis based on the viral genome for a tested input sequence, including DENV sequences. The second database is the Dengue virus genotyping database^2^ (Yamashita et al., 2013), which provides a summary table containing the DENV serotype/genotype, year and country of collection and accession number. There are many other DENV databases; however, no other sites provide summarized genotype information. The Dengue Virus Resource^3^ facilitates the retrieval of DENV sequences deposited in GenBank according to serotype, disease symptom, host, region/country, genome region, and collection and/or release data (Resch et al., 2009). DENVirDB^4^ provides sequence information and computationally curated information of dengue viral proteins (Asnet et al., 2014). DENVDB focuses on the dengue virus sequence database for keyword searches (no publication: http://proline.bic.nus.edu.sg/denvdb/). Finally, the Dengue Virus Portal is a sequence collection with metadata (no publication: https://www.broadinstitute.org/annotation/viral/Dengue/Home.html).

Here, we constructed the website Dengue Genographic Viewer (DGV), which presents DENV information based on the genotype and epidemiological data by using the geographic tool Google Maps^©^ to update the recent dissemination of DENV genotypes from a global perspective.

## Materials and methods

### Construction of the DENV database

The DENV genotype database was constructed as follows: (1) all accessible DENV nucleotide sequences were collected; (2) the complete sequences of each protein region (C, prM, E, NS1, NS2A, NS2B, NS3, NS4A, NS4B, and NS5) were extracted from the sequences; (3) a blastn homology search was performed against the genotype database and the genotype of the most homologous sequence was assigned; (4) and the genotype data were stored in a database using SQLite (https://www.sqlite.org/).

1. The DENV nucleotide sequences were downloaded from the NCBI database using key words (“Dengue virus”[porgn:__txid12637]).
2. A blastx homology search was performed to detect nucleotide regions that corresponded to each mature DENV protein; nucleotide regions that exhibited more than 85% sequence coverage to the protein were used for the subsequent analysis.
3. To reduce the time required to obtain the most homologous sequences in the genotype database, we reduced the number of sequences used in the blast search by clustering highly homologous sequences. We performed a uclust search (Edgar, 2010) against the nucleotide sequences of each protein region and selected one representative sequence for each homologous sequence group with a clustering threshold of 99% identity; then, the representative sequences were subjected to a homology search against the genotype database. The original genotype database was constructed according to the method proposed by previous report (Yamashita et al., 2013). Briefly, the representative sequences were aligned by using the mafft (Katoh and Standley, 2014) program and Neighbor joining (NJ) phylogenetic trees were constructed using the MEGA5 program (Tamura et al., 2011). The genotype of each gene was assigned manually according to the previous genotype database (Yamashita et al., 2013).
4. Sequence ID, country/region and year of collection were extracted from the deposited GenBank data and integrated into the SQL database by using an in house Perl script.

The above processes except for the original database construction are performed automatically every night to update the recent DENV database.

### Interactive viewer using web interface

We implemented a set of viewer applications on DGV by using Google Maps^©^, which shows the data in a temporal and spatial manner. One application presents the geographical distribution of each DENV genotype on the map in a user-specified time span. Another option is a homology search program that searches for the most homologous DENV sequence in the DGV database and show the geographical positions of closely related sequences on the map. The other interface shows the sources of imported dengue cases on the map, according to the Infectious Agents Surveillance Report (IASR), Japan (http://www.nih.go.jp/niid/en/iasr-e.html). This set of applications is available at the DGV web site (https://gph.niid.go.jp/geograph/dengue/content/genomemap).

## Results and discussion

### Distribution of DENV genotypes

On March 4, 2016, DGV included a total of 10,514 DENV sequences, which consisted of 3872, 3414, 2309, and 919 DENV serotype-1, -2, -3, and -4 sequences, respectively (Table 1). Some genotypes have been abundantly sequenced and deposited in the public database, whereas other genotypes have rarely been sequenced (i.e., DENV-1 genotype II was reported in only seven records from 1960 to 2012, DENV-4 genotype III was also reported in only seven records from 1997 to 2001, and DENV-3 genotype IV has not been reported since 1977). These rare genotypes may have become minor populations or may be undergoing a silent transmission cycle (Lanciotti et al., 1994; Chen and Vasilakis, 2011; Santiago et al., 2012).

**Table 1:** **Number of DENV records (March 4, 2016)**.

**Serotype**	**Genotype**	**Records on public database**	**Total sequences of each serotype**
**DENV-1**			
	I	2508	
	Ib	13	
	II	7	
	III	6	
	IV	407	
	V	879	
	Recombinant	49	
	Lab strain	3	3872
**DENV-2**			
	American	101	
	Asian-American	1245	
	Asian I	858	
	Asian II	105	
	Cosmopolitan	1042	
	Sylvatic	26	
	Recombinant	35	
	Unknown	2	3414
**DENV-3**			
	I	326	
	II	742	
	III	1192	
	IV	5	
	V	34	
	Recombinant	9	
	Unknown	1	2309
**DENV-4**			
	I	295	
	IIA	223	
	IIB	385	
	III	7	
	Sylvatic	6	
	Recombinant	3	919

**Figure 1:**
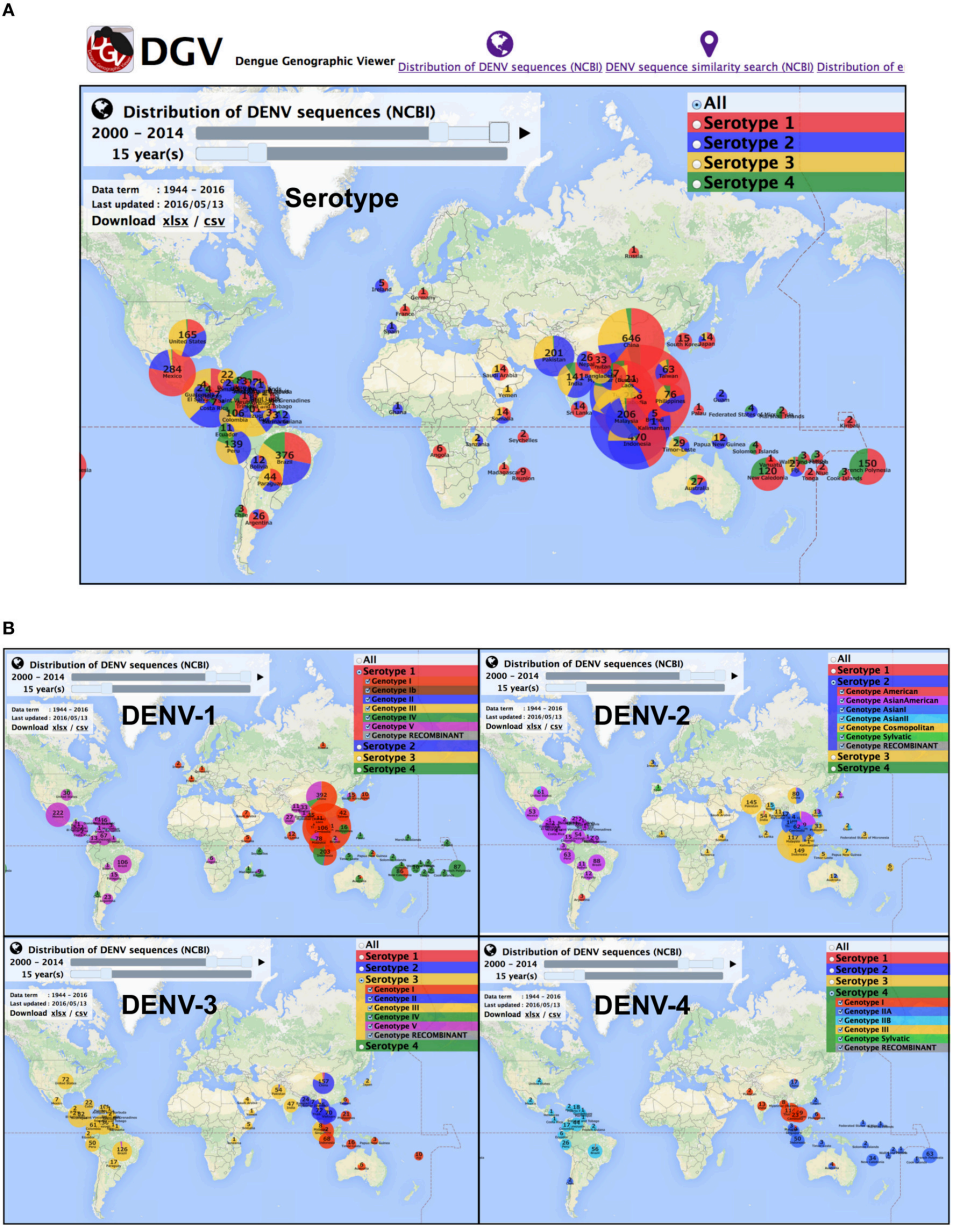
**Overview of DGV for serotype distribution for all DENV (A) and genotype distribution for each serotype (B) in 2000–2014**.

Fifteen years' worth of data from 2000 to 2014 for all serotypes showed that DENV sequences were primarily reported from South to Southeast Asia, Central to South America, and the countries of Oceania (Figure 1A). Some biases in DENV serotype compositions were observed in several countries. For instance, the dominant serotypes were DENV-1 and -2 in Mexico, DENV-1 and -4 in Polynesian countries with the exception of Fiji, and DENV-2 and -3 in Pakistan. In contrast, all serotypes were sampled in Brazil and Thailand.

**Figure 2:**
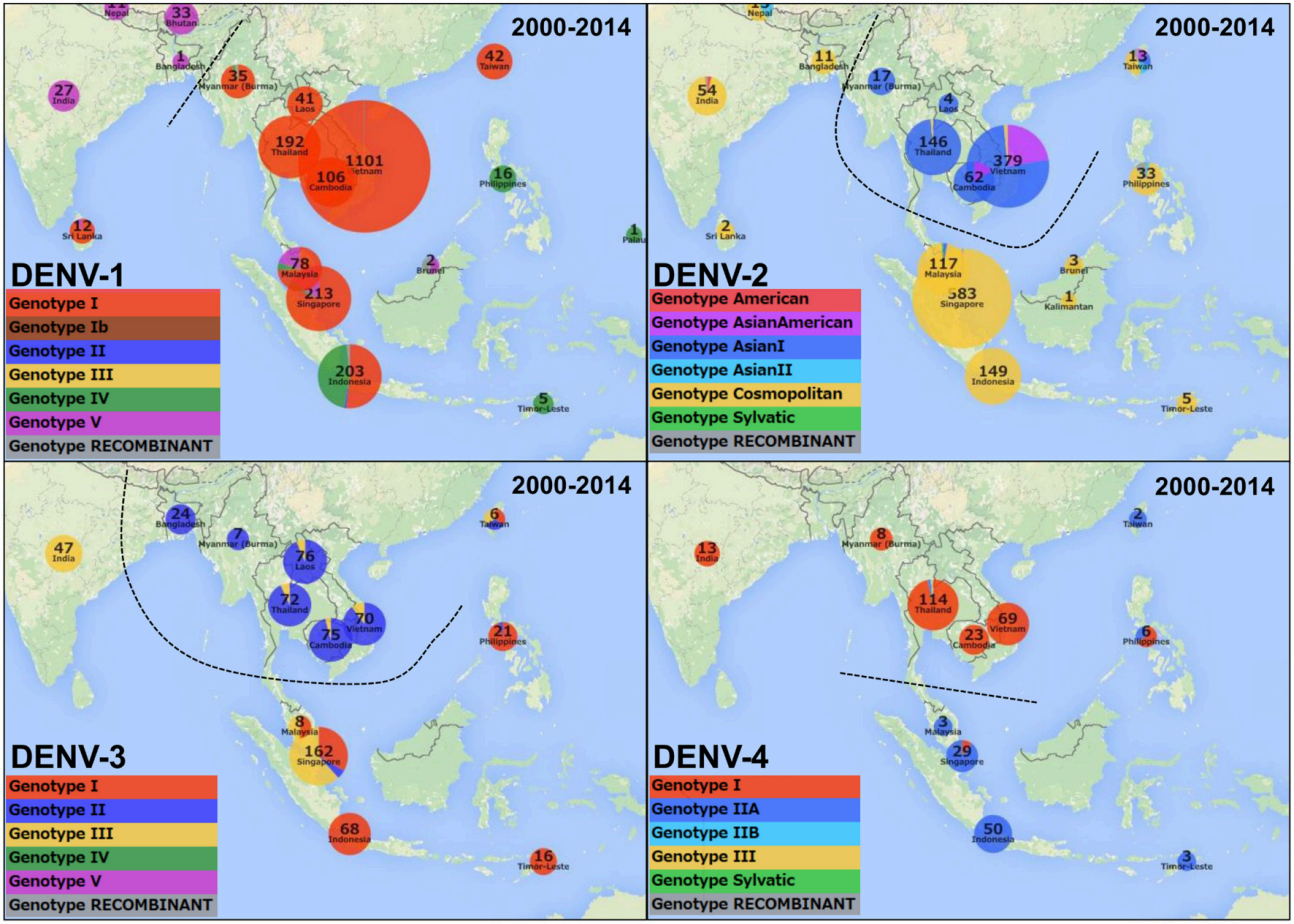
**Genotype distribution for each DENV serotype in Asia in 2000–2014**. Possible borderline of genotype distribution in each serotype is shown as dash line.

Intriguingly, when focusing on the genotype instead of the serotype, the data from 2000 to 2014 showed at least three potential geographical genotype distribution border lines in Asia (Figures 1B,2). The first border is between the American continents and other regions (Figure 1B), the second is located between Bangladesh and Myanmar for the genotype distributions of DENV-1 and -2 and India and Myanmar for DENV-3, and the third is located between Indochina and the Malay Peninsula (Figure 2). There seem to be differences in the DENV-1 and -3 distributions between Malaysia, Singapore and Indonesia; however, the border line is not clear because Malaysia and Indonesia consist of many islands and share Kalimantan Island and the deposited sequence data do not specify the original island isolation site. Although, some boundaries are not clear, these boundaries are roughly conserved among all serotypes except for the Bangladesh-Myanmar border line for DENV-4, suggesting potential barriers against the vector mosquitos' movements or human activities between the countries.

**Figure 3:**
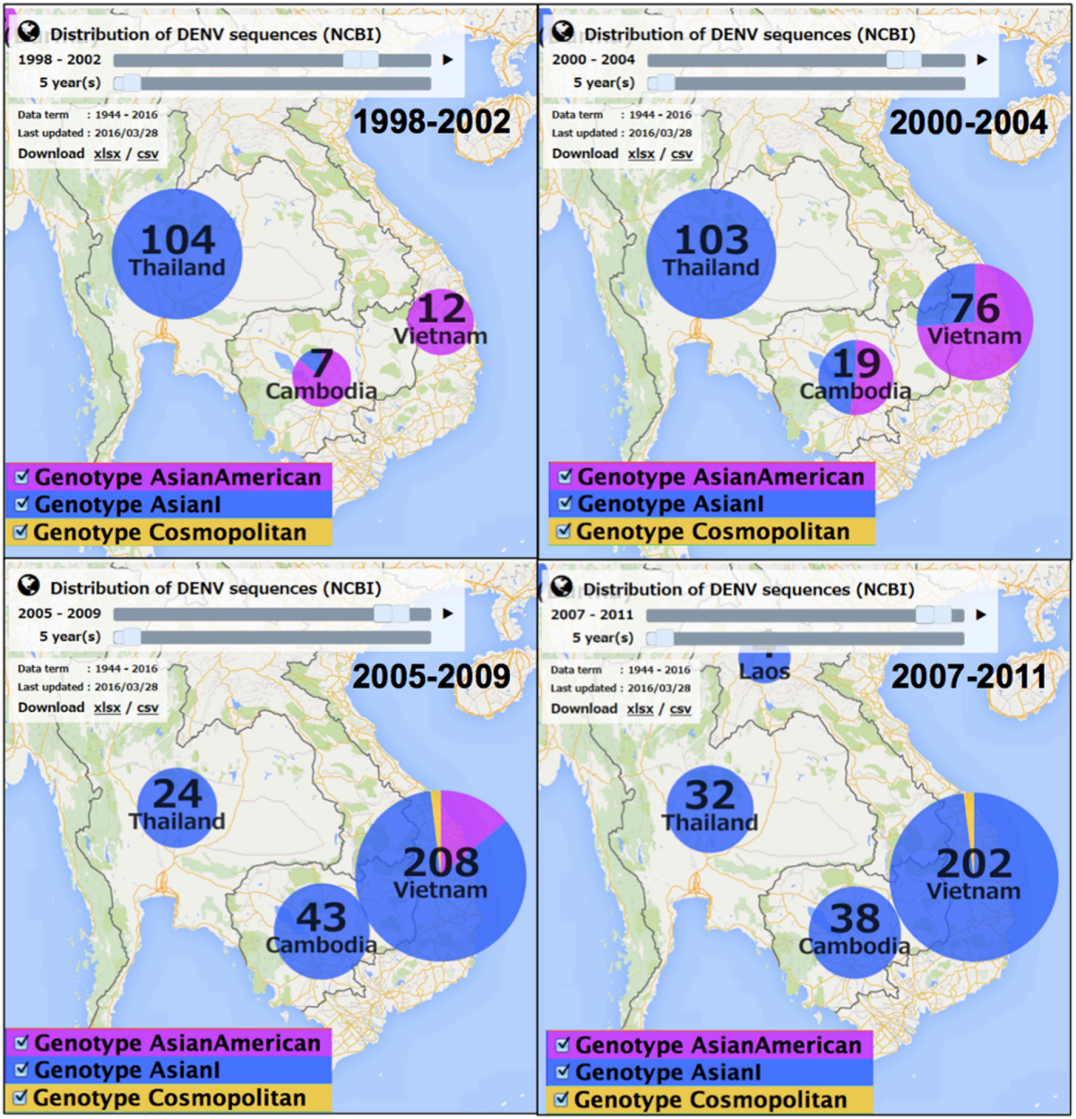
**Genotype distribution of DENV-2 in Indochina from 1998 to 2011**. The movie of the genotype distribution in 1986–2014 can be downloaded as Movie S1.

We also found a timeline change in the predominant genotypes. From 1998 to 2007, the dominant genotype in Asia was Cosmopolitan, although India-Pakistan-Sri Lanka and Southeast-Oceania belonged to different lineages (Khan et al., 2013). The major genotypes in the Indochina countries were different from those of the other Asian countries; genotype Asian I was predominant in Thailand, whereas genotype Asian American was predominant in Cambodia and Vietnam (Figure 3 and Movie S1). From 2001, Asian I increased in Cambodia and Vietnam until finally in 2007 Asian I became the predominant genotype in Indochina. The genotype Asian I viruses in Thailand seemed to be widely disseminated into Vietnam via Cambodia but did not reach Malaysia and Bangladesh (Figure 2). Thus, the Asian American genotype was replaced by Asian I in Cambodia and Vietnam between 1998 and 2011. This example also suggests the idea of genotype transition, which probably reflects the mosquito vector habitat and human activities in the Indochinese Peninsula.

DGV currently does not support the prediction of Dengue epidemics, because number of deposited sequence data does not always reflect the actual number of events, in addition, it takes long time to be a public sequence through isolation, sequencing, and publication.

### DENV sequence similarity search

DGV provides a search engine for the assignment of the DENV serotype, genotype, and origin country according to the most homologous sequence on the basis of a blastn search against the DENV database. The search results are shown as text and are also plotted through Google Maps^©^. Subsequently, the query sequence is divided into mature protein regions and displayed with a serotype/genotype assignment. The homology search results and the divided nucleotide sequences in fasta format can be downloaded.

**Figure 4:**
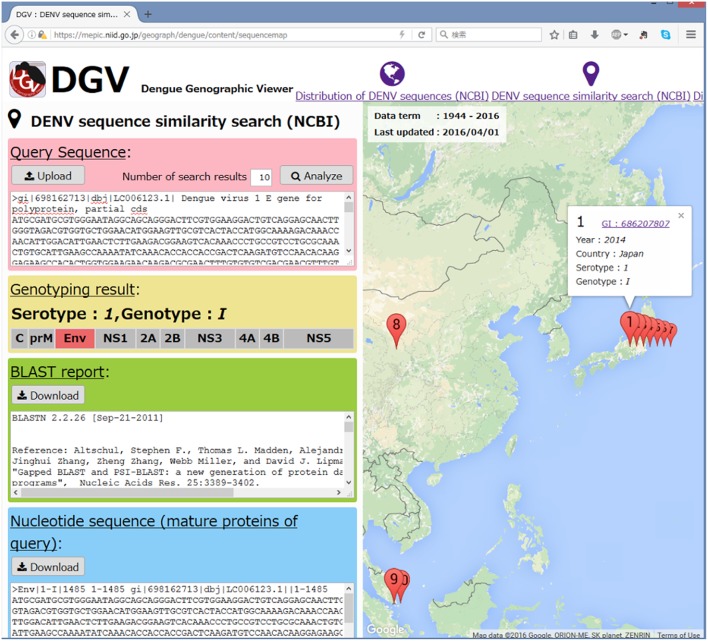
**A screenshot of the DENV sequence similarity search**. An Env sequence derived from an autochthonous case in Japan (LC006123 or gi: 698162713) was used as a sample query. The query was assigned as the Env region of DENV-1 genotype I.

Here, we present an example similarity search for an Env sequence derived from an autochthonous case in Japan (LC006123 or gi: 698162713). DGV assigned the sequence as the Env region of the DENV-1 genotype I and identified homologous sequences from Japan, China, Singapore and Indonesia. These results are consistent with those from a previous study (Figure 4; Kutsuna et al., 2015).

### Distribution of entrants infected with DENV

**Figure 5:**
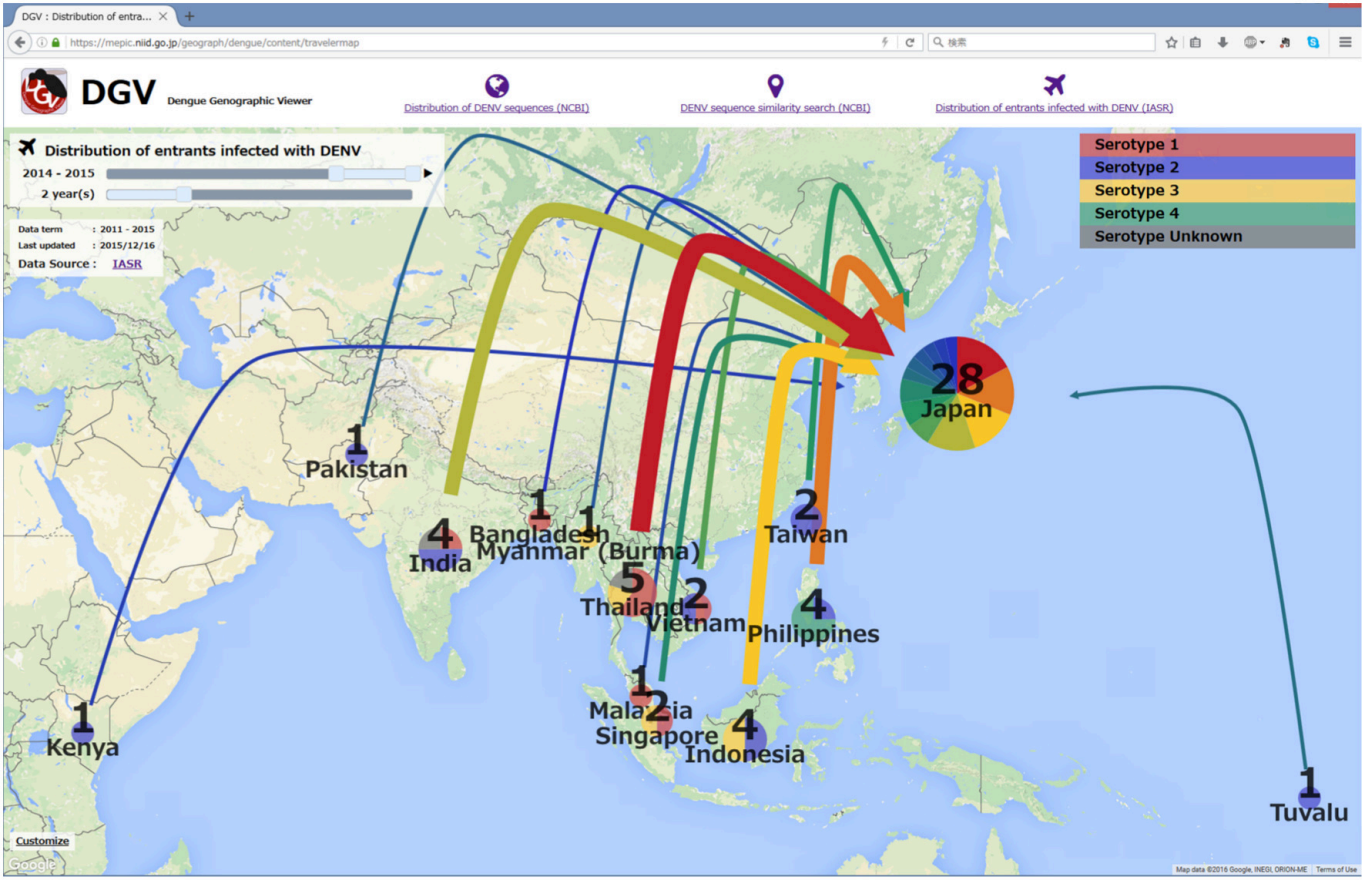
**A screenshot of the distribution of entrants infected with DENV, which shows the cumulative number of infected entrants from 2014 to 2015 based on the IASR**.

To aid in visualizing the source countries of dengue infection cases imported to Japan, the number of annual imported cases was also mapped on Google Maps^©^. The serotype (but not genotype), year, and visiting country/area are also indicated based on the Infectious Agents Surveillance Report (IASR), which releases monthly data and information obtained from prefectural and municipal public health institutes and quarantine stations to the public (Figure 5).

## Author contributions

AY performed the experimental design, participated in the analysis and drafted the manuscript. TS1 implemented the application, performed the data collection, constructed the original genotype database, and participated in the analysis. TS2, KK, and TT reviewed the application and participated in the discussion. MK contributed to the experimental design, performed the analysis and drafted the manuscript. All authors read and approved the final manuscript.

## Funding

This work was supported by a grant for Research on Emerging and Re-emerging Infectious Diseases (H25 Shinko-Ippan-015/H26 Shinko-Gyosei-Shitei-002) from the Ministry of Health, Labor and Welfare, Japan, and was also supported by the Research Program on Emerging and Re-emerging Infectious Diseases (15fk0108011h0003 and 15fm0108022h0001) from the Japan Agency for Medical Research and Development, AMED. This work was also partially supported by JSPS KAKENHI Grant Number 15K08488.The funders had no role in the study design, data collection and analysis, decision to publish, or preparation of the manuscript.

### Conflict of interest statement

The authors declare that the research was conducted in the absence of any commercial or financial relationships that could be construed as a potential conflict of interest.

## Footnotes

^1^ViPR (Virus Pathogen Database and Analysis Resource): http://www.viprbrc.org/brc/home.spg?decorator=flavi_dengue

^2^Dengue virus genotyping database: http://www.gen-info.osaka-u.ac.jp/~uhmin/genotyping/index.html

^3^Dengue Virus Resource: https://www.ncbi.nlm.nih.gov/genome/viruses/variation/dengue/

^4^DEnvirDB: http://www.ladydoakcollege.edu.in/denvirdb/index.php

## Supplementary material

Movie S1 **Genotype transition of DENV-2 in Asia from 1986–1990 to 2010–2014**.

## Abbreviations

DENV: Dengue virus
C: capsid protein
prM: premembrane protein
E: envelope glycoprotein
NS: non-structural protein
NCBI: national center for biotechnology information
IASR: Infectious Agents Surveillance Report.
